# NCX1 disturbs calcium homeostasis and promotes RANKL-induced osteoclast differentiation by regulating JNK/c-Fos/NFATc1 signaling pathway in multiple myeloma

**DOI:** 10.1007/s10238-022-00905-1

**Published:** 2022-10-17

**Authors:** Tingting Li, Dongbiao Qiu, Qingjiao Chen, Apeng Yang, Junmin Chen, Zhiyong Zeng

**Affiliations:** 1 Department of Hematology, The First Affiliated Hospital of Fujian Medical University, 20 Chazhong Road, Fuzhou, 350005 Fujian People’s Republic of China; 2 Department of Blood Transfusion, The First Affiliated Hospital of Fujian Medical University, Fuzhou, People’s Republic of China; 3 Fujian Key Laboratory of Laboratory Medicine, Fuzhou, People’s Republic of China

**Keywords:** NCX1 channel, Calcium, Multiple myeloma, Osteoclast differentiation

## Abstract

Although several types of calcium channels abnormalities have been shown to promote myeloma bone disease (MBD), the relationship between Na^+^/Ca^2+^ exchanger 1 (NCX1) and MBD remains unexplored. Here, we examined the role of NCX1 in the development of multiple myeloma (MM), with a special focus on the underlying effects involved osteoclast differentiation. Firstly, we detected NCX1 protein highly expressed in BM tissues of MM patients, and its expression was positively correlated with serum calcium and the percentage of BM CD138^+^ cells. In vitro, NCX1 suppression with the inhibitor KB-R7943 reduced cell viability of MM cells and caused apoptosis. Extracellular high Ca^2+^ environment increased the level of intracellular Ca^2+^ in MM cells through gating the calcium influx, with subsequently promoting the expression of NCX1 and osteoclastogenesis-related genes (receptor activator of nuclear factor-κB (RANKL), nuclear factor of activated T cell cytoplasmic 1 (NFATc1), and proto-oncogene Fos (c-Fos). This phenomenon could be reversed by KB-R7943 or calcium chelation. Furthermore, NCX1 overexpression in MM cells accelerated osteoclastogenesis, while NCX1 knockdown or suppression resulted in the opposite effect. Mechanistically, we further investigated the related mechanisms of NCX1 regulating osteoclast differentiation using RNA sequencing, western blotting and Enzyme linked immunosorbent assay, and found that NCX1 modulated osteoclast differentiation in MM though JNK/c-Fos/NFATc1 signaling pathway. In conclusion, the Ca^2+^/NCX1-mediated signaling participates in the osteoclasts-myeloma cell interactions, which represents a promising target for future therapeutic intervention in MBD.

**Supplementary Information:**

The online version contains supplementary material available at 10.1007/s10238-022-00905-1.

## Introduction

Multiple myeloma (MM) is a plasma cell malignancy, which has the second highest incidence rate of hematological malignancies [1]. About 70%-80% of MM patients have osteolytic lesions, known as myeloma bone disease (MBD), which is often accompanied by hypercalcemia [2]. Previous studies have shown that elevated cellular calcium is linked to the hyperactivity of osteoclasts (OC), and accelerates bone destruction and resorption in myeloma through MM-OC interaction [3]. Since calcium permeable channels on the plasma membrane are critical in regulating intracellular and extracellular Ca^2+^, their abnormal expression and function are correlated with the development and maintenance of MM phenotypes [4, 5]. Consistently, we summarized that several calcium channels were involved in osteoclast differentiation and prognosis in MM [6]. Therefore, plasma membrane calcium channels involved in high Ca^2+^-induced osteoclast differentiation in MM, which is worthy of further investigation since they may be new potential drug targets for MBD therapy.

The sodium/calcium exchanger (NCX/SLC8A) is a bidirectional plasma membrane calcium transport system, generally transporting one Ca^2+^ ion out of the cell with the influx of three Na^+^ ions [7]. Nevertheless, in pathological settings, the reverse mode of operation was shown to be predominant preferentially, in which three Na^+^ ions extruded, one Ca^2+^ transported into the cells [8]. The three different NCX isoforms (NCX1/SLC8A1, NCX2/SLC8A2 and NCX3/SLC8A3) participate in the maintenance of cellular Ca^2+^ balance [9]. As the most important subtype, NCX1 gene is found in a wide range of mammalian cells, and it plays an important role in the development of cancer [10]. At present, researchers have linked the occurrence and development of several malignancies, including leukemia, to elevated intracellular calcium([Ca^2+^]i) levels caused by NCX1 [11, 12]. However, it has not been investigated how NCX1 channel plays a role in the development of MM and osteoclast differentiation.

In this study, we revealed the overexpression of NCX1 in MM and its effect on the viability of myeloma cells. In addition, we found that the activation of NCX1 by extracellular high calcium microenvironment increased the osteoclastic activity, indicating that NCX1-induced calcium influx of myeloma cells activates JNK/c-Fos/NFATc1 pathway and modulates RANKL-induced osteoclast differentiation. Altogether, this study on the interaction between OC and MM cells modulated by Ca^2+^/NCX1 may give a novel direction for the prevention and therapy of MBD.

## Methods and materials

### Cell lines and tissues

Four MM cell lines (MM.1S, RPMI8226, KMS11, and U266) and monocytic osteoclast progenitor cell line (RAW264.7) were purchased from Procell (Wuhan, China). MM cell lines and RAW264.7 cells were incubated in RPMI-1640 or DMEM medium (Meilunbio, China), which contained 10% FBS (ExCell, China), penicillin and streptomycin. 5% CO_2_ at 37 °C was used to culture all cells. From September 2019 to January 2021, paraffin sections of bone marrow samples of 24 MM patients and 7 iron deficiency anemia (IDA) patients were obtained from the First Affiliated Hospital of Fujian Medical University. And the percentage of CD138 + cells in the bone marrow of MM patients was obtained from the pathology report. Primary plasma cells were isolated from bone marrow specimens of patients with MM and healthy donors using anti-CD138 MicroBeads (Miltenyi, Germany) and immediately frozen in − 80 °C until the subsequent extraction of RNA. The Ethics Committee of the First Affiliated Hospital of Fujian Medical University approved this study. According to the Declaration of Helsinki, informed consent was obtained.

### Viral infection

To obtain cell lines stably expressing NCX1/SLC8A1, RPMI8226 and KMS11 cells were transfected with HBLV-h-SLC8A1-3xflag-PURO or HBLV-PURO virus (Hanheng Biotechnology, Shanghai, China) (multiplicity of infection = 50) for 48 h and chose with 0.5–1 μg/ml puromycin for 4 days. Lentiviruses containing shRNA targeting human NCX1(Shanghai Genechem Co., LTD, China) was transfected into RPMI8226 or KMS11 cells to obtain NCX1 knockdown MM cells. The sequences of the shRNAs were as follows: shRNA1, gcTAGGATTCTGAAGGAACTT. shRNA2, gcCATCTTCTAAGACTGAAAT. shRNA3, ccTGAGATTCTCCTTTCAGTA. Compared with shRNA1 and shRNA2, shRNA3 was found to be more efficient in knockdown of NCX1 by validation (data not shown), and in subsequent experiments, shRNA3 was selected for knockdown of NCX1 in MM cells.

### Immunohistochemistry (IHC)

In this study, BM specimens were prepared from paraffin-embedded blocks, and thin slips of 4 μm were cut and submitted to immunohistochemistry (IHC) of anti-NCX1 (Cat. No. ab2869, Abcam, UK) at 4 °C overnight. NCX1 was detected with goat anti-mouse secondary antibody (1:200; maxim, China) and visualized by diaminobenzidine (DAB). Negative controls contain secondary antibody only. Microscopic observation was performed after counterstaining with Harris hematoxylin (Meilunbio, China) for 30 min. ImagePro Plus was used to measure the yellow optical density sum (IOD SUM), area (area of measurement area). Finally, the mean density (mean density = (IOD SUM) /area) was calculated.

### Quantitative real-time PCR (qRT-PCR)

To detect the expression of NCX1 mRNA in plasma cells isolated from bone marrow of patients with MM and healthy donors, RNA was extracted using Lab aid 820 nucleic acid extraction Mini reagents (Xiamen Zhishan Biological Technology Co., Ltd.), and then synthesized into cDNA using HiScript III 1st Strand cDNA Synthesis Kit (+ gDNA wiper) (Nanjing Vazyme Biotech Co., Ltd). For NCX1 gene, qRT-PCR was performed using the following single primer/probe sets: forward primer: 5′-GGAACATCAGTGCCAGACACATT-3′; reverse primer: 5′-TGACGTTACCTATGGAGGCG-3′. The fluorescent probe, 5′-6-FAM-CAGCCACCCAGGACCAGTATGCAG-BHQ1-3′. The Rnase gene was chosen as a control (housekeeping) gene to evaluate the amount and amplification of cDNA. The qRT-PCR primer/probe set for the Rnase gene was as follows: forward primer:5′-AGATTTGGACCTGCGAGCG-3′; and reverse primer: GAGCGGCTGTCTCCACAAGT-3′. The fluorescent probe, 5′-6-FAM -TTCTGACCTGAAGGCTCTGCGCG-BHQ1-3′. The primers and probes were synthesized by Sangon Biotech (ShangHai, China). PCR conditions: 2 min at 37 °C followed by 5 min at 95 °C and 40 two-step amplification cycles consisting of 10 s at 95 °C followed by 40 s at 55 °C. qRT-PCR was performed on the Thermo Fisher 7500 PCR machine (Applied Biosystems, USA) with Taq Pro HS U + Probe Master Mix (Nanjing Vazyme Biotech Co., Ltd).

We also extracted RNA from MM cells using TRIzol (Life technologies, USA) based on the manufacturer’s protocol. RNA was synthesized into cDNA using the EasyScript One-Step gDNA Removal and cDNA Synthesis SuperMix kit (Beijing TransGen Biotech, China). The manufacturer’s procedure: 10 min at 25 °C, 15 min at 42 °C, then 5 s at 65 °C. RNA levels were quantified by qRT-PCR using the LightCycler 480II system (LightCycler 480II, Roche) with 95 °C for 30 s, 95 °C for 5 s, then annealing temperature for 30 s for 43 cycles in total. RNA level assay were determined by the 2^−ΔΔCt^, and β-actin as a control. SYBR Green (Beijing TransGen Biotech, China) was used as a double-stranded DNA specific dye. The following primary sequences were obtained from Sangon Biotech (ShangHai, China): β-actin forward primer: 5′-GGCATCCACGAAACTACCTT-3′; reverse primer: 5′-CGGACTCGTCATACTCCTGCT-3′; NCX1 (forward primer: 5′-TGTGCATCTCAGCAATGTCA- 3′, reverse primer: 5′-TTCCTCGAGCTCCAGATGTT- 3′); RANKL (forward primer: 5′-AAGGAGCTGTGCAAAAGGAA-3′; reverse primer: 5′-CGAAAGCAAATGTTGGCATA-3′). Relative expression of target genes was determined using the 2^−ΔΔCt^ method. The data was normalized to expression of the housekeeping gene. Expression levels were then normalized to those in the corresponding control groups.

### Western blotting

We extracted total protein using radioimmunoprecipitation assay (RIPA) strong buffer (Meilunbio, China) with newly added protease inhibitors. Nuclear and plasma proteins were extracted through the manufacturer’s protocol for the Mammalian Nuclear and Cytoplasmic Protein Extraction Kit (Beijing TransGen Biotech, China). Cell lysates were separated using 7.5–12% PAGE Gel Rapid Preparation Kit (Shanghai Epizyme Biomedical Technology Co., Ltd) and transferred to PVDF membranes (Merck Millipore Ltd, Germany); After blocking with 5% nonfat milk for 1–2 h, the membranes were incubated with the corresponding primary antibodies overnight at 4 °C. Primary antibodies used are as follows: NCX1 (Cat. No. ab177952, Abcam, UK), NFATc1 (Cat. No.YT5381, ImmunoWay Biotechnology, China), c-Fos (Cat. No.YT0884, ImmunoWay Biotechnology, China), RANKL (Cat. No.YT5404, ImmunoWay Biotechnology, China), p-JNK (Cat. No. YP0157, ImmunoWay Biotechnology, China), JNK (Cat. No.YT2440, ImmunoWay Biotechnology, China), p-P38(Cat. No. AP0526, ABclonal Technology, China), P38(Cat. No. A4771, ABclonal Technology, China), p-ERK (Cat. No.BS1112, Bioworld Technology, Inc. USA), ERK (Cat. No.BS4621, Bioworld Technology, Inc. USA), β-actin (Cat. No. HC201-01, Beijing TransGen Biotech, China), Histone H3 (Cat. No. YM3038, ImmunoWay Biotechnology, China). The next day, the blots were blotted with HRP-conjugated secondary antibodies (Beijing TransGen Biotech, China) and then visualized on an ECL detection system (BIO-RAD). Results were quantified by densitometry using the software ImageJ software.

### Cell proliferation assay

Cell Counting Assay Kit (CCK)-8 (Meilunbio, China) was used to assess the proliferative activity of MM cells. Cells were inoculated into a 96-well plate (1 × 10^4^ cells per well) for different time (0, 24, 48, 72 and 96 h), followed by incubation at 37 °C. A microplate reader (Tecan Infinite F50; Tecan Group, Ltd.) was used to measure the absorbance at 450 nm after incubation with 10 µl CCK-8 reagent for 1 h.

### Colony formation assay

The long-term proliferation of cells could be detected by their ability to form colonies. Firstly, seeded 400 MM cells per 6-well plate. After 14 days, we removed the cell culture medium and gently washed the cell culture plate containing the colonies with PBS, then fixed them for 20 min using 3.7% formaldehyde (Meilunbio, China). The colonies were washed with PBS again, stained with crystal violet solution (Meilunbio, China) for 10 min and their numbers were measured (relative clonality = number of clones/average number of clones in the control group).

### Cell apoptosis assay

MM cells were treated with FITC-conjugated Annexin V (Bioworld Technology, Inc. USA) and PI (Bioworld Technology, Inc. USA) to label apoptotic and dead cells, respectively. The staining step was performed according to the manufacturer's protocol. Then, the apoptosis rate of cells was analyzed on a flow cytometer (BD Biosciences, USA) within 1 h.

### Calcium influx assay

The [Ca^2+^]i concentration was detected by Fluo-4 AM (Beyotime, China), a cell-permeable fluorescent calcium indicator. Briefly, cells were cultured with RPMI-1640 for 48 h and washed 3 times in Ca^2+^-free D-Hanks Balanced Salt Solution (Ca^2+^-free HBSS). Then, the cells were incubated for 40 min at 37 °C in the dark with 3 μmol/l Fluo-4 AM. They were then washed 3 times in Ca^2+^-free HBSS to remove extracellular Fluo-4 AM. Replace the solution with Ca^2+^-containing HBSS 1 min before the test. The fluorescence intensity of [Ca^2+^]_i_ was detected by flow cytometry, excited with 488 mm, and the fluorescence signal was collected by the FLI-H fluorescence channel. 1 × 10^4^ cells were collected, and the [Ca^2+^]_i_ concentration was expressed as the mean fluorescence.

### Osteoclast differentiation and MM cell 50% conditioned medium (CM)

RAW264.7 cell suspension (1.5 × 10^4^ cells/well) was plated into 24-well plates and cultured with MM cell 50% CM which refers to 1.5 × 10^4^ cells/24 well of MM cells treated with RPMI-1640 medium (containing 1% FBS) for 48 h, and then DMEM medium (containing 10% FBS) was added in the ratio of 1:1 for the culture of RAW264 0.7 cells [13, 14]. TRAP staining and ghost ring peptide staining were performed after 6–7 days.

### ELISA assay

Quantitative levels of human RANKL in the conditioned media (CM) of MM were detected by ELISA kit following the manufacturer's instructions (BOYAN Biotech, China).

### TRAP staining

Osteoclast differentiation of RAW264.7 co-cultured with MM CM was assessed after 6 days of culture conditions by the detection of TRAP activity, according to the procedure provided by the manufacturer (Acid-phosphatase leukocyte staining kit; Sigma–Aldrich, USA) and observed by optical microscopy. Mature OC are defined as multinucleated TRAP^+^ cells that contain more than 3 nuclei. As control, RAW264.7 cells were incubated with 100 ng/mL sRANKL (PeproTech, USA) and 25 ng/mL macrophage-colony stimulating factor (M-CSF) (PeproTech, USA).

### Fluorescence staining of F-actin rings

We tested the fibrous actin (F-actin) ring for osteoclast function. According to the procedure provided by the manufacturer (share-bio, China), 3.75% formaldehyde was used to fix cells for 15 min after washing in PBS. 0.5% Triton X-100 (Hangzhou Fude Biotechnology Co., Ltd.) was added for 10 min to increase permeability, and then the cells were treated with red 594-phalloidin-conjugated working solution for 20 min. And nuclei were stained with 4′,6-Diamidino-2-phenylindole dihydrochloride (DAPI) (Beyotime, China). The formation of F-actin loop was observed and quantified by fluorescence microscopy.

### Gene Set Enrichment Analysis (GSEA)

To explore related signaling pathways, GSEA was performed on the RNA-seq results of the normal control group and the KB-R7943 treatment group or the NCX1 knockdown group. Gene sets “c2.cp.kegg.v2022.1.Hs.symbols.gmt” were obtained from the MSigDB. The R package clusterProfiler was applied for KEGG-related GSEA analysis.

### Statistical analysis

GraphPad Prism 8.0 was used for the statistical analysis. All experimental results were from at least three replicates. Data were expressed as the mean ± standard deviation (SD). Student's t tests were used to analyze statistical differences between two groups, while one-way ANOVA was used to compare three or more groups. Pearson's correlation analysis was used to examine the association between NCX1 and CD138^+^ cells (%) or serum calcium. Statistical significance was defined as P values less than 0.05.

## Results

### Increased NCX1 expression in human MM BM tissues and cells, and its expression is positively correlated with serum calcium

**Fig. 1:**
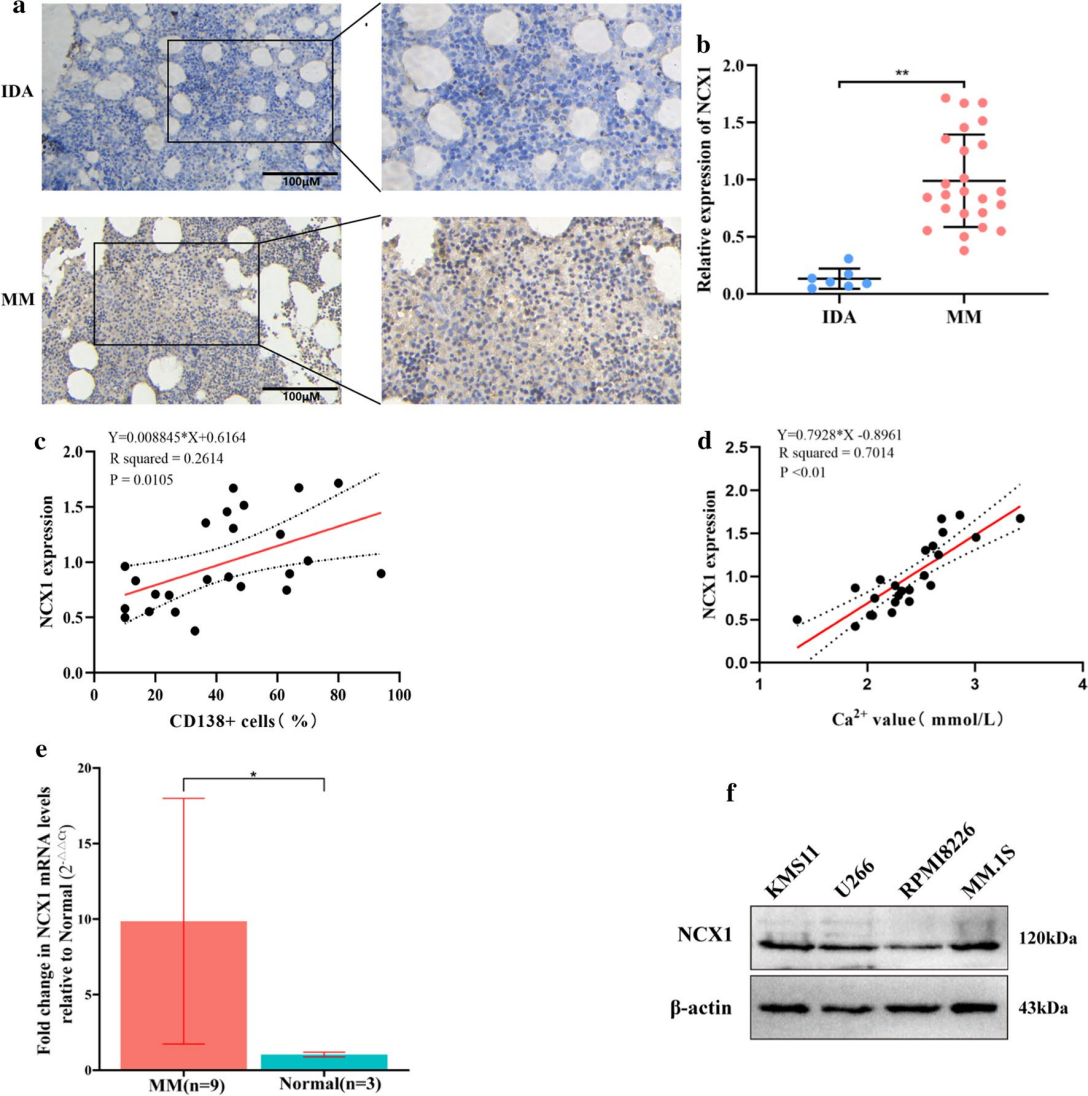
NCX1 is highly expressed and correlation with calcium levels in multiple myeloma (MM). **a** Representative image of immunohistochemical staining on NCX1 protein levels in bone marrow (BM) samples from iron deficiency anemia (IDA) (*n* = 7) and MM patients (*n* = 24) (Scale bar = 100 μm). **b** Summary data of NCX1 protein expression by immunohistochemical staining (**p < 0.01). **c** Analysis of the correlation between NCX1 protein expression and BM CD138^+^ cells in MM patients (*n* = 24) (*p < 0.05). **d** Analysis of the correlation between NCX1 expression (detected by immunohistochemical staining) and serum calcium in MM patients (*n* = 24) (**p < 0.01). **e** qRT-PCR analysis of NCX1 mRNA expression in CD138^+^ plasma cells of bone marrow from normal donors (n = 3) and MM patients (n = 9). Relative expression was calculated using the 2^−ΔΔCt^ method with RNase as the reference gene. The NCX1 mRNA levels of the MM cases were normalized to that of the “Normal.” Data were presented as mean ± standard deviation (SD) (*p < 0.05). **f** NCX1 expression in MM cell lines (KMS11, U266, RPMI8226 and MM.1S) was examined by western blotting

Abnormal expression of calcium channels is often associated with cancer progression. Immunohistochemical analysis showed that the expression level of NCX1 in BM tissues of patients with MM (*n* = 24) was significantly higher than that of patients with IDA (*n* = 7) (Fig. 1a, b). And its expression was positively related to the percentage of CD138^+^ cells in BM (Fig. 1c). Since MBD patients are often accompanied by hypercalcemia [2], we further found that NCX1 expression was positively related to serum calcium (Fig. 1d). The results of qPCR analysis showed that the expression level of NCX1 mRNA in CD138^+^ plasma cells of bone marrow of patients with MM (*n* = 9) was significantly higher than that of normal donors (*n* = 3) (Fig. 1e).

Then, we assessed NCX1 protein levels in 4 MM cell lines (KMS11, U266, RPMI8226, MM.1S). According to western blotting, all these MM cell lines expressed NCX1 (Fig. 1f), so it can be used for subsequent experiments. Collectively, these results suggest that NCX1 was indeed highly expressed in MM cells, and might play an important role in the pathogenesis of MBD.

### Inhibition of NCX1 inhibits MM cell proliferation and induces apoptosis

**Fig. 2:**
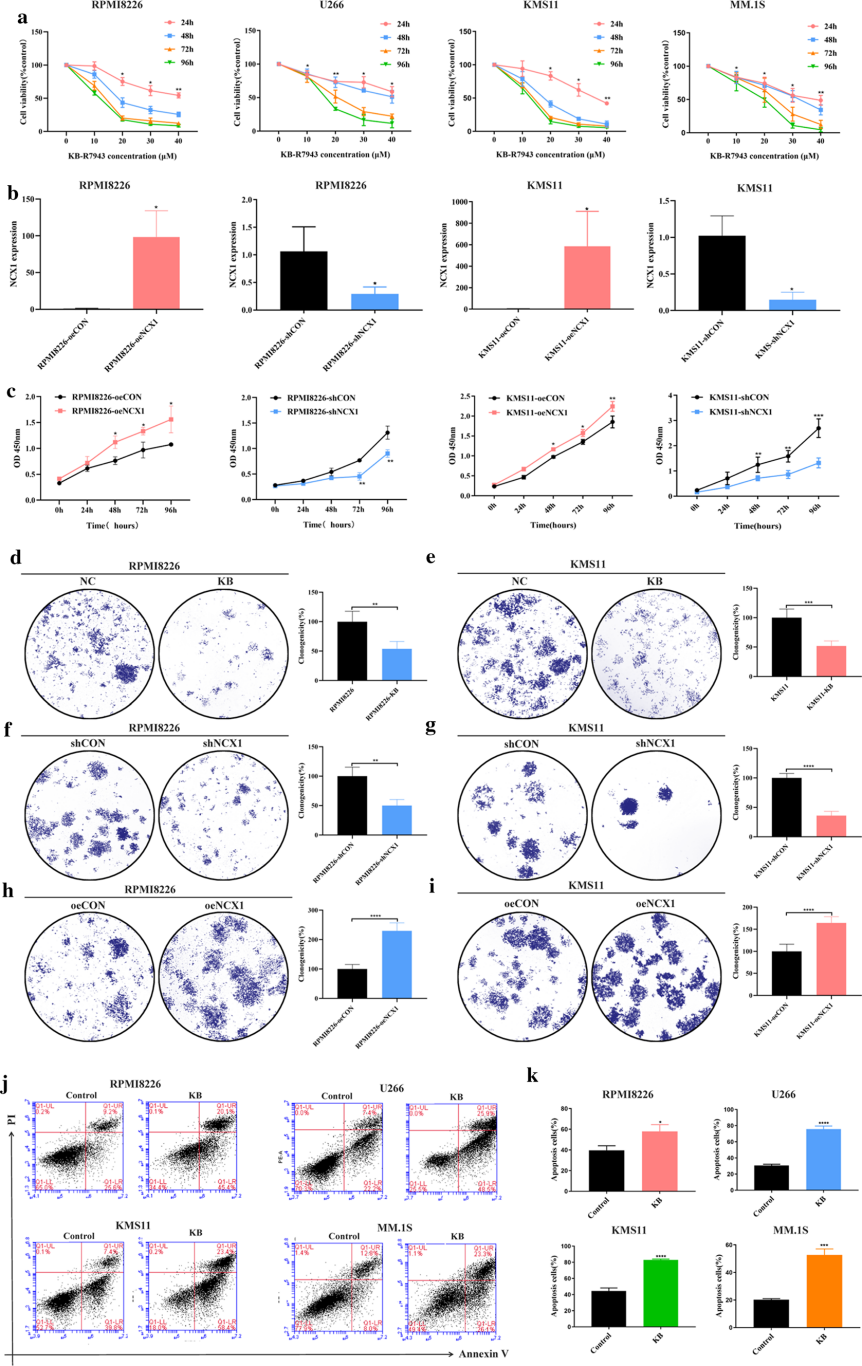
Effects of NCX1 on cell proliferation, clonal formation and apoptosis of multiple myeloma (MM) cells. **a** RPMI8226, KMS11, U266 and MM.1S cells were exposed to different concentrations (10 μM,20 μM,30 μM,40 μM) of KB-R7943 for 48 h. Cell proliferation was measured by CCK-8 assay (*p < 0.05, **p < 0.01, *n* = 3). **b** Relative expression of NCX1 detected by qRT-PCR in RPMI8226 and KMS11 cells after transfection with the lentiviral system of NCX1-overexpression or NCX1-knockdown (*p < 0.05, *n* = 3). **c** MM cells proliferation in NCX1-overexpressed or knockdown RPMI8226 cells and in NCX1-overexpressed or knockdown KMS11 cells, compared with control vector (*p < 0.05, **p < 0.01, ***p < 0.001, *n* = 3). **d-i** Images of representative clonal formation (left) and summary data (right), revealing the effect of KB-R7943 on clonogenicity in RPMI8226 (**d**) and KMS11 cells (**e**), the effect of NCX1 knockdown on clonogenicity in RPMI8226 (**f**) and KMS11 cells (**g**), and the effect of NCX1 overexpression on clonogenicity in RPMI8226 (**h**) and KMS11 cells (**i**) (**p < 0.01, ***p < 0.001, ****p < 0.0001, *n* = 4). **j** Cell apoptosis detected by flow cytometry of RPMI8226, KMS11, U266 and MM.1S exposed to KB-R7943 for 48 h, and summary data(**k**) (*p < 0.05, ***p < 0.001, ****p < 0.0001, *n* = 3)

To further determine if NCX1 was a contributing factor to the development of MM, we exposed 4 MM cell lines (RPMI8226, KMS11, U266, MM.1S) to KB-R7943, an inhibitor of reverse-mode NCX1, for 24 h, 48 h, 72 h and 96 h. Compared with the control group, KB-R7943 significantly suppressed the proliferation of MM cell lines in a concentration- and time-dependent manner (Fig. 2a). Then, we used lentiviral system to overexpress or knockdown NCX1 expression in RPMI8226 cells and KMS11 cells (Fig. 2b). Results of CCK-8 showed that cell proliferation was increased in NCX1 overexpressed (named as oeNCX1) cells, but decreased in NCX1 knockdown (named as shNCX1) cells (Fig. 2c), which was consistent with the result of KB-R7943. Parallelly, the clonal formation assay revealed the promotive role of NCX1 on clonal formation in MM cells (Fig. 2d–i).

Moreover, Annexin V/PI staining revealed that a significant increase in early and late apoptosis was observed in all 4 MM cell lines where NCX1 inhibition was achieved with KB-R7943 (Fig. 2j, k). These results demonstrate that NCX1 is crucial to MM proliferation and apoptosis, and might be an oncogene for humans with MM.

### High extracellular calcium ([Ca^2+^]_o_) induces calcium influx, and promotes the expression of NCX1 and osteoclastogenesis-related genes in MM cells

**Fig. 3:**
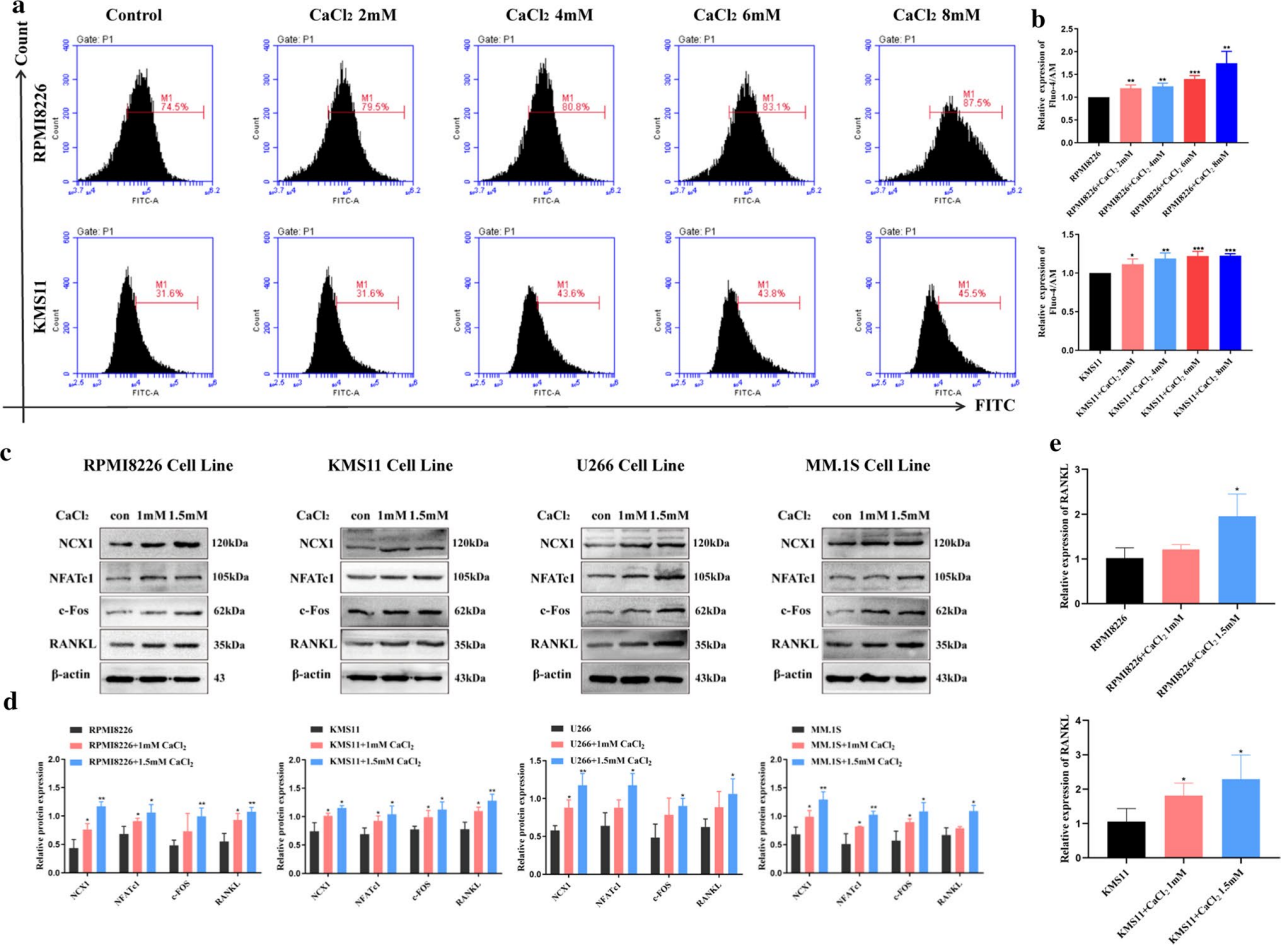
Effects of high concentration of [Ca^2+^]_o_ on the expression of NCX1 and osteoclastogenesis-related genes in multiple myeloma (MM) cells. **a** Representative images of 2 mM, 4 mM, 6 mM and 8 mM CaCl_2_-induced [Ca^2+^]_i_ in RPMI8226 and KMS11 cells. [Ca^2+^]_i_ was measured by flow cytometry and cells were incubated with Fluo-4 AM, a cytoplasmic Ca^2+^ specific indicator. **b** Summary data are displayed as a bar graph. (*p < 0.05, **p < 0.01, ***p < 0.001, *n* = 3). **c** Western blot analysis of NCX1, RANKL, NFATc1 and c-Fos protein expression in 4 MM cell lines exposed to different concentrations (1 mM and 1.5 mM) of CaCl_2_ for 48 h, and statistical data (**d**) (*p < 0.05, **p < 0.01, *n* = 3). **e** mRNA levels of RANKL detected by qRT-PCR in RPMI8226 cells and KMS11cells exposed to CaCl_2_ (1 mM and 1.5 mM) (*p < 0.05, *n* = 4)

In MM, hypercalcemia is a poor prognostic factor closely associated with bone resorption and bone destruction [15]. As shown above, NCX1 protein expression was positively correlated with serum calcium concentration in newly diagnosed MM patients. To better understand the role of NCX1 in MM hypercalcemia, we simulated the extracellular hypercalcemia environment by adding a certain concentration of CaCl_2_ to cell culture medium. Firstly, we evaluated the influences of [Ca^2+^]_o_ on [Ca^2+^]_i_ levels. MM cells (RPMI8226, KMS11) were exposed to CaCl_2_ at different concentrations for 1 min and analyzed by flow cytometer. As demonstrated in Fig. 3a, b, [Ca^2+^]_o_ could increase [Ca^2+^]_i_ levels after stimulation of 2, 4, 6, and 8 mM CaCl_2_, respectively. Then, we investigated the expression of NCX1 protein in response to increased [Ca^2+^]_o_ concentration. The results showed that [Ca^2+^]_o_ significantly increased NCX1 expression (Fig. 3c, d), suggesting that [Ca^2+^]_o_ can be implicated in the regulation of the expression of NCX1.

Due to the close relationship between hypercalcemia and OC hyperactivity [3], we also further analyzed how [Ca^2+^]_o_ influenced osteoclast differentiation in MM. OC are multinucleated cells differentiated from monocyte or macrophage cells in response to RANKL [16]. Moreover, the osteoclast differentiation process is governed by many OC marker genes, including NFATc1 and c-Fos. When MM cells were stimulated with CaCl_2_, the levels of RANKL were significantly increased in a dose-dependent way (Fig. 3c–e). Altogether, these data demonstrate that high concentration of [Ca^2+^]_o_ indeed stimulate NCX1 expression and enhance the expression of genes (RANKL, NFATc1 and c-Fos) related to osteoclastogenesis.

### NCX1 inhibition with KB-R7943 disturbs calcium influx and reverses the high [Ca^2+^]_o_ induced increase in NCX1 and osteoclastogenesis-related genes expression in MM cells

**Fig. 4:**
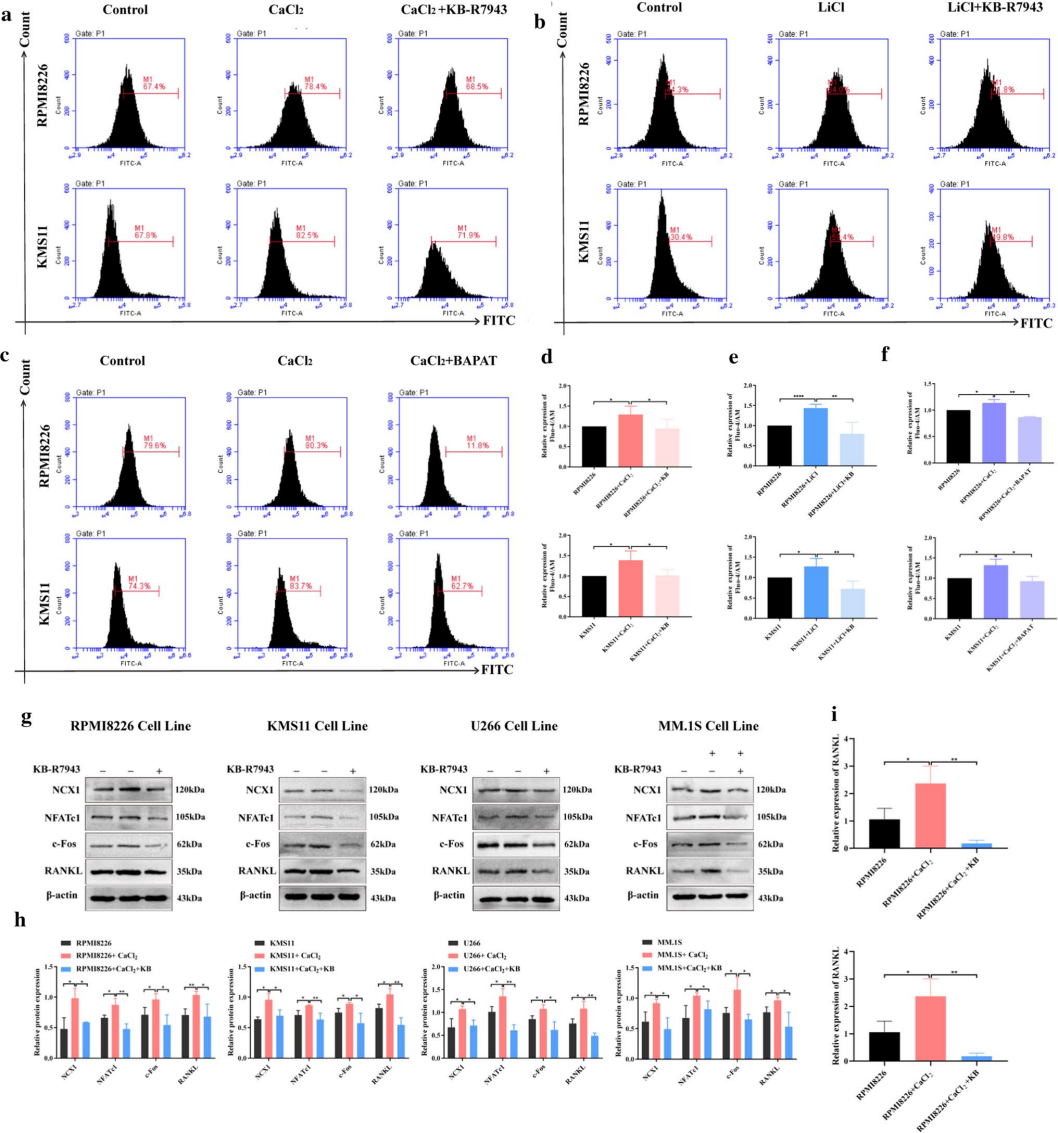
Effects of NCX1 inhibition on calcium influx and osteoclastogenesis-related genes expression in multiple myeloma (MM) cells. **a–f** [Ca^2+^]_i_ was assessed using Fluo-4 AM and flow cytometry analysis. RPMI8226 and KMS11 cells were exposed to CaCl_2_ (1.5 mM) in the absence or presence of KB-R7943 (20 µM) (**a**) or LiCl (10 µM) (**b**) or BAPAT (10 µM) (**c**) for 48 h, and [Ca^2+^]_i_ data was summarized as a bar graph (**d**–**f**) (*p < 0.05, **p < 0.01, *n* = 3). **g** Western blot analysis of NCX1, NFATc1, c-Fos, and RANKL protein expression in MM cells (RPMI8226, U266, KMS11, MM.1S) exposed to CaCl_2_ (1.5 mM) in the absence or presence of KB-R7943 (20 µM) for 48 h, and statistical data (**h**) (*p < 0.05, **p < 0.01, *n* = 3). **i** mRNA levels of RANKL detected by qRT-PCR in MM cells (RPMI8226 and KMS11) exposed to CaCl_2_ (1.5 mM) in the absence or presence of KB-R7943 (20 µM) for 48 h (*p < 0.05, **p < 0.01, *n* = 3)

According to flow cytometry analysis, KB-R7943 pretreatment significantly inhibited the increase in [Ca^2+^]_i_ level in MM cells induced by CaCl_2_ when compared with the CaCl_2_ group (Fig. 4a). Then, using HBSS solution that NaCl was substituted by LiCl (10 mM), we found that compared with the control group, LiCl increased the levels of [Ca^2+^]_i_, and KB-R7943 reversed calcium influx (Fig. 4b), supporting the assumption that NCX1 had the function of transporting Ca^2+^ in MM cells.

Next, RPMI8226 and KMS11 cells were exposed to BAPTA-AM (a cell-permeable calcium chelator) to test whether the decrease in [Ca^2+^]_i_ levels after NCX1 inhibition was dependent on calcium consumption. Based on flow cytometry analysis, Fig. 4c indicates that BAPAT reversed calcium influx induced by CaCl_2_. These results suggest that NCX1 inhibition might disturb calcium homeostasis in MM cells and exert the same effects as BAPAT. And we also confirmed that BAPAT can inhibit the viability of MM cells while increasing the apoptosis of MM cells (Additional file 1).

Furthermore, western blotting showed that MM cells cultured with KB-R7943 for 48 h could reverse the increase in RANKL, NFATc1 and c-Fos protein expression induced by high [Ca^2+^]_o_ (Fig. 4g, h). As expected, qRT-PCR showed that KB-R7943 could reverse the expression of RANKL in MM cells stimulated by CaCl_2_ (Fig. 4i). Of note, BAPAT interfered with calcium homeostasis and was also able to reverse the high [Ca^2+^]_o_-induced elevation of osteoclastogenesis-related protein expression in MM cells (Additional file 1). Based on these findings, NCX1 appears to regulate the expression of osteoclastogenesis-related genes by disturbing calcium homeostasis.

### Role of NCX1 in promoting MM cells-induced osteoclastogenesis

**Fig. 5:**
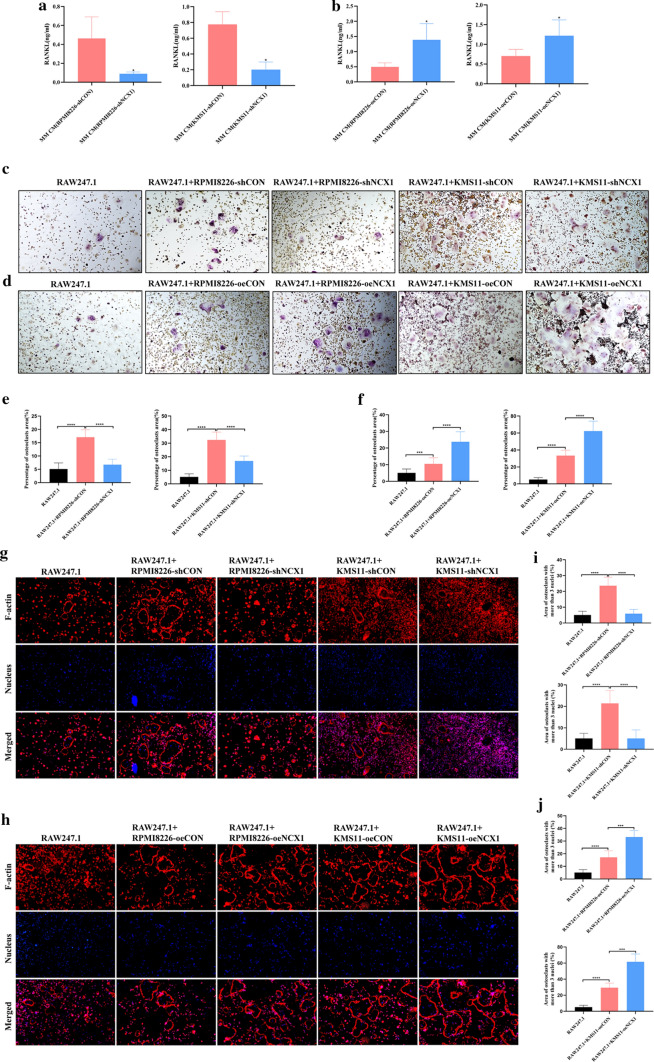
Effects of NCX1 on the secretion of RNAKL and osteoclastogenesis of multiple myeloma (MM) cells. RAW264.7 cells were cultured alone or in the conditioned media (CM) collected from the control (shCON or oeCON), shNCX1 and oeNCX1 RPMI8226 and KMS11 cell lines for 6 days, and then treated with 100 ng/ml RANKL and 25 ng/ml M-CSF. **a**, **b** RANKL protein level in MM CM was measured by ELISA. shCON or shNCX1 in RPMI8226 and KMS11(**a**), oeCON or oeNCX1 in RPMI8226 and KMS11 (**b**). **c**, **d** Osteoclasts (OC) were detected by TRAP staining, and summary data about percentage of OC area (**e**, **f**). shCON or shNCX1 in RPMI8226 and KMS11 (**c**, **e**), oeCON or oeNCX1 in RPMI8226 and KMS11 **(d**,** f**). **g**, **h** Representative images of F-actin rings. Cells fixed and then stained with 594-phalloidin and DAPI. Red, F-actin; Blue, nuclei. shCON or shNCX1 in RPMI8226 and KMS11 (**g**), oeCON or oeNCX1 in RPMI8226 and KMS11 (**h**). **i**, **j** Quantitative analyses of F-actin rings showing the area of OC with more than 3 nuclei. shCON or shNCX1 in RPMI8226 and KMS11 (**i**), oeCON or oeNCX1 in RPMI8226 and KMS11 (**j**)

After confirming that NCX1 inhibition reversed the increased expression of osteoclastogenesis-related genes induced by high [Ca^2+^]_o_, we next investigated the effect of NCX1 on osteoclastogenesis in MM. Since murine macrophage cell line RAW264.7 has been widely used in osteoclast differentiation research [17], we cultured RAW264.7 alone or co-cultured with 50% conditioned media (CM) from shNCX1, oeNCX1 and control (shCON or oeCON) MM cell lines for 6 days, and added 100 ng/ml RANKL and 25 ng/ml M-CSF in media. As illustrated in Fig. 5a, b, the levels of RANKL were decreased in RPMI8226-shNCX1 or KMS11-shNCX1 CM compared to control CM (Fig. 5a). In contrast, the levels of RANKL were increased in RPMI8226-oeNCX1 or KMS11-oeNCX1 CM compared to control CM (Fig. 5b). Interestingly, the number and size of TRAP^+^ OC were increased in co-culturing with MM CM in comparison to RAW264.7 monoculture. Moreover, knockdown of NCX1 in RPMI8226 and KMS11 cells reduced the number and size of TRAP^+^ OC when compared to the corresponding control cells (Fig. 5c, e), but overexpression of NCX1 in RPMI8226 and KMS11 cells increased the number and size of TRAP^+^ OC (Fig. 5d, f). TRAP^+^ OC were not detected in RAW246.7 only incubated with regular media (data shown in Additional file 2).

The F-actin ring is a crucial property of mature OC [18, 19]. After RANKL and M-CSF treatment, phalloidin staining showed that RAW264.7 co-cultured with MM CM formed not only more number but also bigger size of F-actin rings than RAW264.7 alone. Moreover, compared to control CM, knockdown of NCX1 in MM cells decreased F-actin ring number and size (Fig. 5g, i), while overexpression of NCX1 in MM cells increased these parameters (Fig. 5h, j). These results clearly indicate that NCX1 is essential for osteoclastogenesis induced by RANKL in MM.

### NCX1 modulates RANKL-induced osteoclast differentiation though JNK/c-Fos/NFATc1 pathway in MM cells

**Fig. 6:**
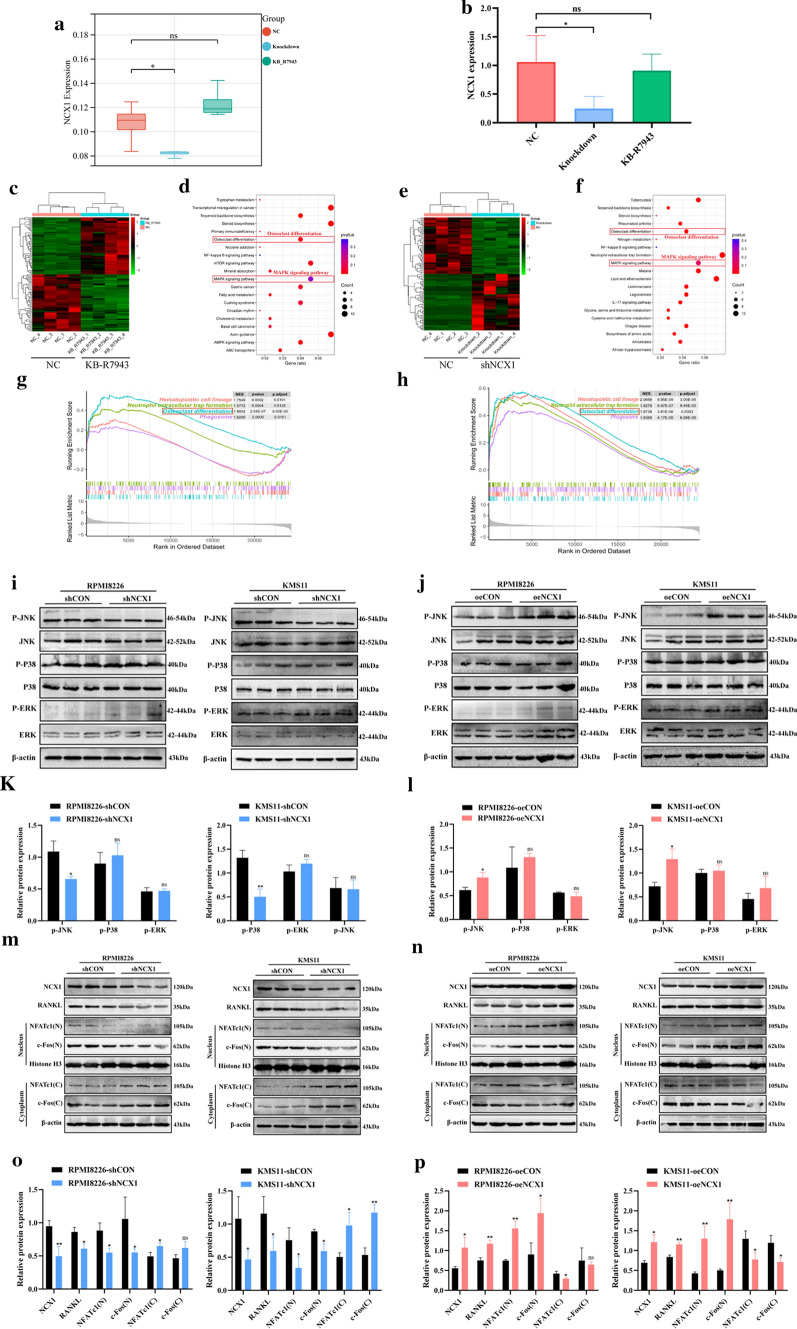
NCX1 modulates RANKL-induced osteoclastogenesis via the JNK/c-Fos/NFATc1 pathway of multiple myeloma (MM). **a, b** NCX1 expression levels of RNA-seq data (**a**) (*p < 0.05, *n* = 4) and NCX1 RNA expression levels detected by qRT-PCR (**b**) (*p < 0.05, *n* = 3). **c-f** Heatmap of RNA-seq data showing significantly different genes before and after using KB-R7943 in KMS11(**c**) or between negative control (NC) and NCX1-knockdown in KMS11 (**e**), and pathway enrichment analysis of RNA-seq data revealed enrichment of two pathways, including osteoclast differentiation and MAPK pathway (**d**, **f**). **g**, **h** The GSEA results showed the top 4 most significantly regulated gene sets in KB-R7943-treated cells (**g**) or NCX1-knockdown cells (**h**), compared with normal control cells, and indicated the NES, p value, p-adjust for each given enriched gene set. **i-l** Western blot analysis of phosphorylated protein expression level of P38, ERK and JNK **(i)** in NCX1 knockdown cells (RPMI8226-shNCX1 and KMS11-shNCX1), compared to their corresponding NC (RPMI8226-shCON and KMS11-shCON) (*p < 0.05, *n* = 3) or (**j**) in NCX1 overexpression cells (RPMI8226-oeNCX1 and KMS11-oeNCX1), compared to their corresponding NC (RPMI8226-oeCON and KMS11-oeCON) (*p < 0.05, *n* = 3). Quantitative analyses of relative ratios of cytoplasmic p-P38/P38, p-ERK/ERK and p-JNK/JNK (**k**, **l**) (*p < 0.05, **p < 0.01, *n* = 3). **m–p** Western blot analysis of NCX1 and RANKL protein expression, and protein levels of NFATc1 and c-Fos in the cytoplasmic and nuclear extracts. shCON or shNCX1 in RPMI8226 and KMS11 cells (**m**). oeCON or oeNCX1 in RPMI8226 and KMS11 cells (**n**). Quantitative analyses of relative ratios of cytoplasmic NFATc1/β-actin, c-Fos/β-actin and nuclear NFATc1/histone H3, c-Fos/histone H3 (**o**, **p**) (*p < 0.05, **p < 0.01, *n* = 3)

Next, we investigated the possible mechanism of how NCX1 regulates osteoclast differentiation in MM. We employed RNA sequencing (RNA-seq) on myeloma cells by KB-R7943 treatment or NCX1 knockdown. The analysis showed that RNA expression level of NCX1 was decreased in NCX1-knockdown MM cells compared with control cells, but not in KB-R7943-treated MM cells (Fig. 6a). This result was consistent with the detection of RNA expression levels of NCX1 by qRT-PCR (Fig. 6b). And KEGG analysis showed that inhibition of NCX1 function or expression was associated with enrichment of osteoclast differentiation-related pathways and MAPK-related pathways (Fig. 6c–f). The results of KEGG-related GSEA analysis in KB-R7943 treatment group and NCX1 knockout group both suggested the osteoclast differentiation pathway was one of the top 4 most significantly regulated gene sets (Fig. 6g, h). MAPK signaling pathway has been implicated in a number of aspects of osteoclastogenesis in previous studies [20, 21]. Therefore, we further examined the phosphorylation status of ERK, JNK and p38 proteins by western blotting. Compared to the corresponding control cells, JNK phosphorylation was markedly decreased in NCX1-knockdown MM cells, but increased in NCX1-overexpression MM cells (Fig. 6i–l). By contrast, ERK phosphorylation status and p38 phosphorylation status were unchanged. Thus, activation of JNK signaling is specifically affected by NCX1 expression.

NFATc1 and c-Fos are not only osteoclast marker genes but also nuclear transcription factors that function in the downstream of JNK signaling [21, 22]. Of note, the above analyses shown in Fig. 4 have revealed that KB-R7943 can significantly reverse the increased expression of NFATc1, c-Fos, and RANKL as a result of high [Ca^2+^]_o_. To verify the impacts of NCX1 on RANKL secretion and NFATc1 and c-Fos activity, we performed western blots to determine whether NCX1 expression level affects RANKL expression, NFATc1 nuclear translocation, and c-Fos nuclear translocation. Compared to the corresponding control cells, NCX1-knockdown in MM cells strongly decreased the expression of RANKL, and blocked nuclear translocation of NFATc1 and c-Fos (Fig. 6m, o). In contrast, NCX1-overexpressed cells exhibited increased levels of RANKL protein and nuclear translocation of NFATc1 and c-Fos (Fig. 6n, p). Data about the mRNA levels of RANKL are shown in Additional file 3. Consistent with previous studies, calcium oscillations can affect RANKL secretion in MM cells by regulating NFAT signaling [23, 24]. In addition, NFATc1 expression has been known to be activated by the binding of c-Fos to its promoter region [25, 26], but osteoclastogenesis of osteoclast precursors without c-Fos can be rescued by NFATc1 [27]. Recently, there is growing evidence that JNK signaling regulates the activity of c-Fos/NFATc1 [28, 29]. Our findings show that NCX1 might be involved in RANKL secretion through the promotion of JNK and c-Fos/NFATc1.

## Discussion

Although numerous new anti-myeloma therapies have significantly improved the survival and prognosis of patients with MM [30, 31], MBD remains a major problem. The interaction between certain cytokines secreted by MM cells and OC is known to play a key role in the pathogenesis of MBD [32]. Here, we investigated the role of NCX1 channel in MM and uncovered how it affects osteoclast differentiation.

According to our results, we have verified that NCX1 is highly expressed in MM, and its expression levels are positively related to the percentage of BM CD138^+^ cells. And elevated levels of NCX1 promoted MM cell proliferation and inhibited apoptosis. Importantly, we demonstrated for the first time that NCX1 overexpression in MM BM tissues was closely correlated with elevated serum calcium. MBD is often accompanied by hypercalcemia, which seriously affects the prognosis of the MM patients [6]. To simulate a high calcium environment in vitro, we added a certain concentration of CaCl_2_ to MM cells culture medium. We found that high calcium upregulated NCX1 channel expression in MM cells, and [Ca^2+^]i levels were mainly increased by stimulating Ca^2+^ influx mediated by NCX1 channel, while the NCX1 inhibitor KB-R7943 almost reverses calcium influx induced by high [Ca^2+^]o. These results suggest a correlation between NCX1 and Ca^2+^ in MM, and [Ca^2+^]i oscillations may be caused by the expression and functional change of NCX1 channel.

MBD is usually the result of enhanced bone resorption due to increased osteoclast activity [33, 34]. In MM, Osteolytic lesions are found only adjacent to intramedullary plasma cell foci or plasma cell tumors, indicating that MM cells may release factors (such as RANKL or IL-6) that trigger the activation of OC [35–37]. In MBD, tumor necrosis factor (TNF) family member RANKL plays a key role in the increased osteoclastogenesis [38, 39]. And it has been reported that high Ca^2+^ can stimulate RANKL secretion and induce osteoclast differentiation in co-cultures without osteoclast differentiation promoting factors [40]. In agreement with these reports, our results revealed that [Ca^2+^]_o_ induced the secretion of RANKL from MM cells, as well as the expression of genes related to osteoclastogenesis such as NFATc1 and c-Fos. However, these can be reversed by NCX1 inhibitor KB-R7943. In addition, NCX1 not only promoted nuclear translocation of NFATc1 and c-Fos, but also increased RANKL-induced osteoclastogenesis in MM-OC co-cultured system. These results suggest that RANKL secretion and osteoclastogenesis-related genes expression are closely related to NCX1 channel in MM.

We provided further experimental data to support our view that NCX1 channel is critical for osteoclast differentiation in MM. The downregulation of NCX1 expression of MM cells attenuated the levels of RANKL in MM CM. In contrast, upregulation of NCX1 expression of MM cells promoted the levels of RANKL in MM CM. Therefore, NCX1 channel seems to play a role as RANKL activator because most calcium channels are usually associated with RANKL secretion [4, 41]. Furthermore, NCX1-knockdown in MM cells decreased the formation of TRAP^+^ OC and F-actin rings in MM-OC co-cultures. In contrast, NCX1-overexpression in MM cells enhanced the formation of both TRAP^+^ OC and F-actin rings. Taken together, our results suggest that aberrant expression of NCX1 channels is associated with the secretion of RANKL, and it plays a critical role in RANKL-induced osteoclastogenesis.

Using RNA-seq to assess NCX1 expression and related signaling pathways, NCX1 RNA expression levels were reduced in NCX1-knockdown MM cells compared to control cells, but not in KB-R7943-treated MM cells. Combined with the above KB-R7943 can inhibit NCX1 protein level and calcium ion transport function, indicating that KB-R7943 mainly affects its function through the protein level. Furthermore, KEGG-related GSEA analysis showed osteoclast differentiation-related pathway was one of the most significantly regulated gene sets after inhibiting NCX1 function or expression in MM cells using KB-R7943 or NCX1 knockdown. The MAPK signaling pathway is closely related to many aspects of osteoclastogenesis. We found that NCX1 regulated the phosphorylation level of JNK protein, but not p38 or ERK by western-blot. Taken together, we discovered for the first time that NCX1, as a Ca^2+^ permeable channel, might exert its reverse operation mode in MM cells, and promote the expression of RANKL in MM cells via JNK/c-Fos/NFATc1 pathway to induced osteoclast differentiation.

## Conclusion

**Fig. 7:**
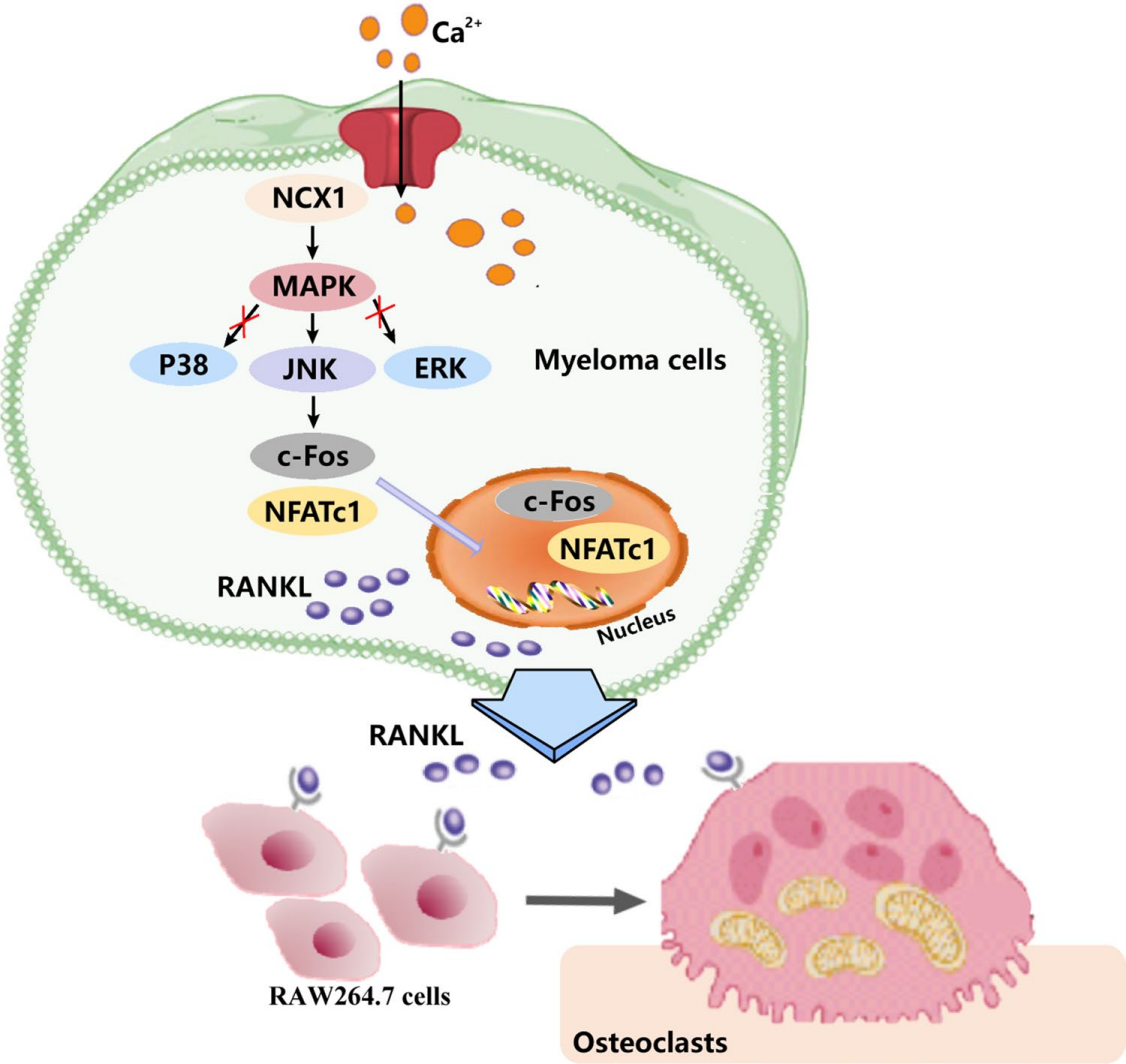
Proposed mechanisms of NCX1-mediated osteoclast differentiation in multiple myeloma (MM). The calcium entry via NCX1 channel causes activation of JNK/c-Fos/NFATc1 signaling pathway and RANKL secretion of MM cells, leading to osteoclast differentiation

In conclusion, our data demonstrate that NCX1 is implicated in MM cell proliferation, apoptosis and osteoclast differentiation. Mechanistically, NCX1 disturbs calcium homeostasis and modulates the excessive secretion of RANKL in MM cells though JNK/c-Fos/NFATc1 pathway, thereby promoting osteoclast differentiation (Fig. 7).

## Supplementary Information

Below is the link to the electronic supplementary material.

Supplementary file1 (DOCX 757 kb)

## Abbreviations

BM: Bone marrow
CM: Conditioned medium
[Ca^2+^]o: Extracellular calcium
DAB: Diaminobenzidine
F-actin: Fibrous actin
GSEA: Gene Set Enrichment Analysis
IHC: Immunohistochemistry
M-CSF: Macrophage-colony stimulating factor
NCX: Sodium/calcium exchanger
NES: Normalized enrichment scores
PBL: Peripheral blood
RNA-seq: RNA sequencing
TNF: Tumor necrosis factor
CCK-8: Cell Counting Assay Kit-8
[Ca^2+^]i: Intracellular calcium
c-Fos: Proto-oncogene Fos
DAPI: 4′,6-Diamidino-2-phenylindole dihydrochloride
GEP: Gene expression profiling
HBSS: D-Hanks Balanced Salt Solution
MBD: Myeloma bone disease
MM: Multiple myeloma
NCX1: Na^+^/Ca^2+^ exchanger 1
NFATc1: Nuclear factor of activated T cell cytoplasmic 1
OC: Osteoclasts
RANKL: Receptor activator of nuclear factor-κB ligand
SD: Standard deviation
TRAP: Tartrate-resistant acid phosphatase
