# Supplementary material for: NCX1 disturbs calcium homeostasis and promotes RANKL-induced osteoclast differentiation by regulating JNK/c-Fos/NFATc1 signaling pathway in multiple myeloma
Source: Clin Exp Med. 2022 Oct 17;23(5):1581–96. doi: 10.1007/s10238-022-00905-1 (PMC10460717; doi:10.1007/s10238-022-00905-1)
Supplement: Supplementary file 1 — Supplementary file1 (DOCX 757 kb) [file 10238_2022_905_MOESM1_ESM.docx]

**Additional file**

**
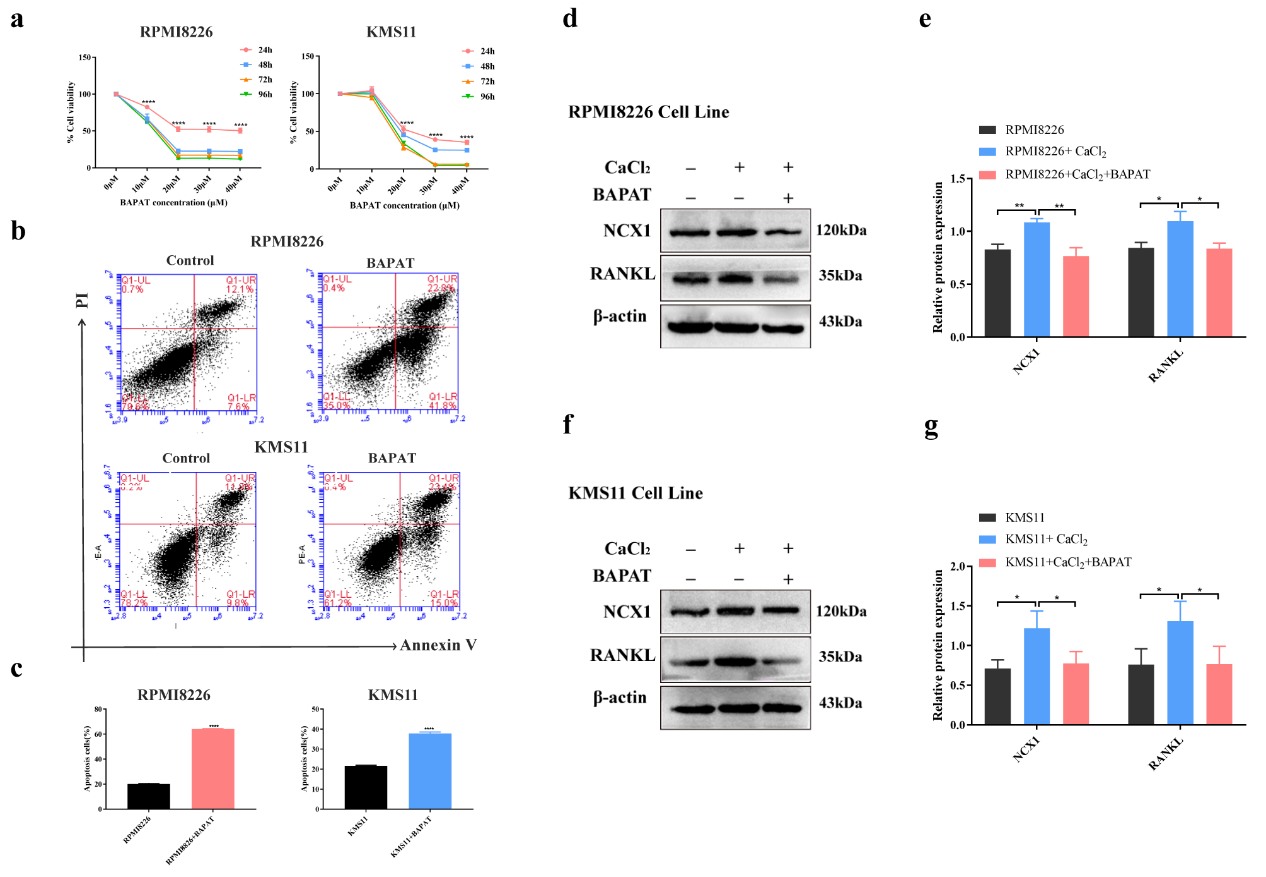
**

**Additional file 1 Effects of BAPAT on cell proliferation, apoptosis and RNAKL protein expression in MM cells** **a** RPMI8226 and KMS11 cells were exposed to different concentrations(10μM,20μM,30μM,40μM) of BAPAT for 48h and cell proliferation was measured by CCK-8 assay (****p<0.0001, n=3). **b** Cell apoptosis of RPMI8226 and KMS11 cells were exposed to BAPAT (10μM) for 48 h by flow cytometry, and summary data(**c**) (****p<0.0001, n=3). **d-g** Western blot analysis of NCX1 and RANKL protein expression of RPMI8226(**d**) and KMS11 cells(**f**) were exposed to CaCl_2_(1.5mM) in the absence or presence of BAPAT (10 µM) for 48 h, and statistical data of RPMI8226(**e**) and KMS11 cells (**g**) (*p<0.05, **p<0.01, ***p<0.001, n=3).


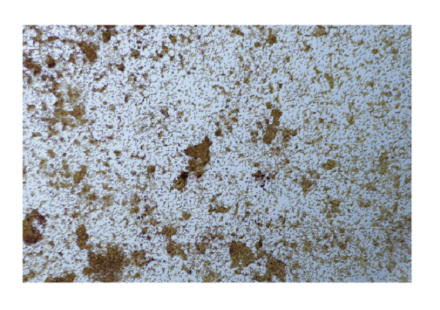


**Additional file 2 TRAP staining of RAW246.7 incubated with regular media (without RANKL and M-CSF treatment)**


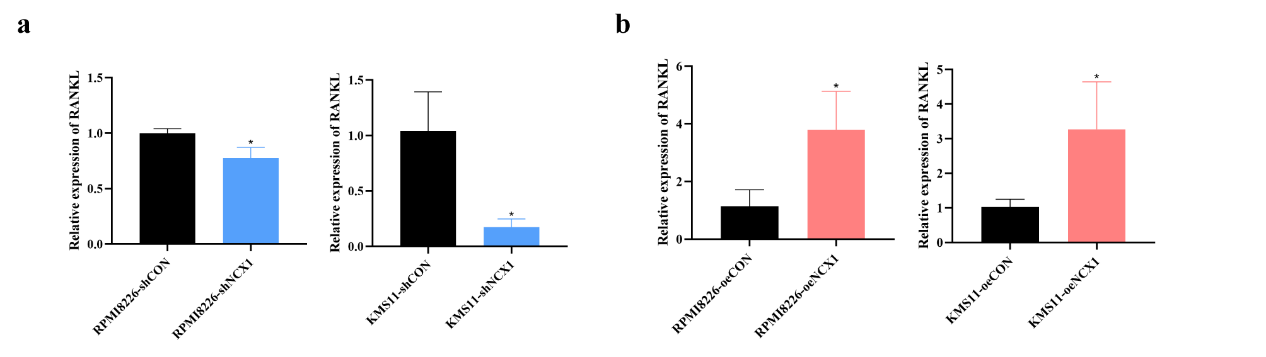


**Additional file 3 Effects of NCX1 on MM cells-induced the expression of RNAKL a** mRNA levels of RANKL detected by qRT-PCR in NCX1 knockdown cells (RPMI8226-shNCX1 and KMS11-shNCX1), compared to their corresponding NC (RPMI8226-shCON and KMS11-shCON) (*p<0.05, n=3). **b** mRNA levels of RANKL detected by qRT-PCR in NCX1 overexpression cells (RPMI8226-oeNCX1 and KMS11-oeNCX1), compared to their corresponding NC (RPMI8226-oeCON and KMS11-oeCON) (*p<0.05, n=3).
